# A Case of a High Lateral Acute Myocardial Infarction Caused by Occlusion of a First Diagonal Branch With Subtle Electrocardiogram Changes and Negative Cardiac Biomarkers at Presentation

**DOI:** 10.7759/cureus.109857

**Published:** 2026-05-29

**Authors:** Naoki Onoda, Kimito Ishikawa

**Affiliations:** 1 Department of Cardiology, Ryugasaki Saiseikai Hospital, Ryugasaki, JPN

**Keywords:** diagonal branch occlusion, high lateral myocardial infarction, occlusion myocardial infarction, percutaneous coronary angioplasty, subtle electrocardiogram change

## Abstract

A 61-year-old man presented to our hospital with chest pain that had begun 50 minutes earlier. On arrival, an electrocardiogram (ECG) showed no obvious ST-T change, and troponin T was negative. The next morning, 11 hours after having chest pain, a re-examined ECG showed no obvious ST-T changes compared to the initial ECG, except for T wave inversion in lead aVL, and troponin T became positive. At that time, acute myocardial infarction (AMI) was highly suspected, and an emergent coronary angiogram was performed. Occlusion of the first diagonal branch was revealed, and successful percutaneous coronary angioplasty was performed. The later clinical course was uneventful. We realized that it is important to compare an initial ECG with serial or prior ECGs to be aware of subtle ECG changes and to repeat cardiac biomarker measurements a few hours later, in order not to miss or delay the diagnosis of AMI, when it has not been ruled out.

## Introduction

Acute myocardial infarction (AMI) is characterized by myocardial necrosis resulting from an acute occlusion of a coronary artery. Myocardial salvage by immediate reperfusion therapy is critical to limit myocardial necrosis. It is known that a short time from onset to reperfusion of an occluded coronary artery is associated with better clinical outcomes [1]. In daily practice, physicians sometimes encounter patients with AMI whose initial electrocardiogram (ECG) and cardiac biomarkers are subtle and nondiagnostic. In such cases, diagnosing AMI with subsequent reperfusion therapy could be delayed.

It is known that ECG changes are sometimes subtle in high lateral wall myocardial infarction [2]. This is because there are no leads that directly show this region, and the distance between the electrodes and this region is relatively far, resulting in small potential changes that are less noticeable in an ECG.

Here, we report a case of an AMI caused by occlusion of a first diagonal branch with subtle ECG changes and negative cardiac biomarkers at presentation, resulting in a delayed diagnosis. From this case, we realized that it is important to compare an initial ECG with serial or prior ECGs to be aware of subtle ECG changes and to repeat cardiac biomarker measurements a few hours later in order not to miss or delay the diagnosis of AMI, when it has not been ruled out.

## Case presentation

A 61-year-old man without medical history presented to our hospital with chest pain that had begun 50 minutes earlier while he was taking a bath in the evening.

On arrival, the blood pressure was 110/76mmHg, the heart rate 56/min, the oxygen saturation 98% while he was breathing ambient air, and the temperature 36.0℃. Auscultation of the lungs and heart was normal. An ECG showed a sinus rhythm without obvious ST-T change (Figure 1). Chest radiography showed no abnormality (Figure 2). Laboratory findings are shown in Table 1.

**Figure 1:**
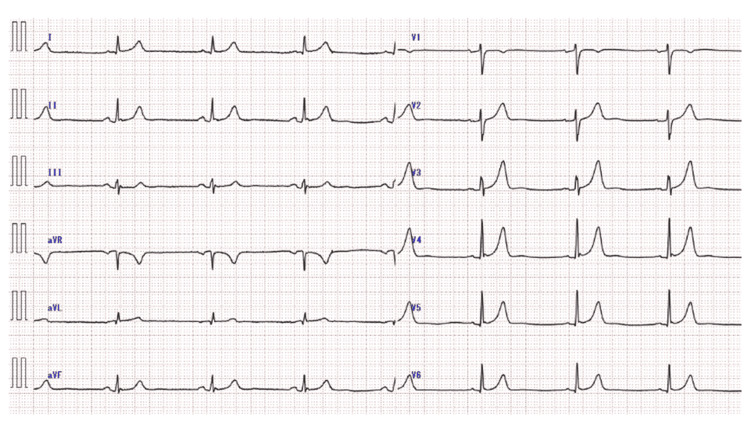
Electrocardiogram at arrival (50 minutes after AMI onset) The initial electrocardiogram showed no obvious ST-T change.  AMI denotes acute myocardial infarction.

**Figure 2:**
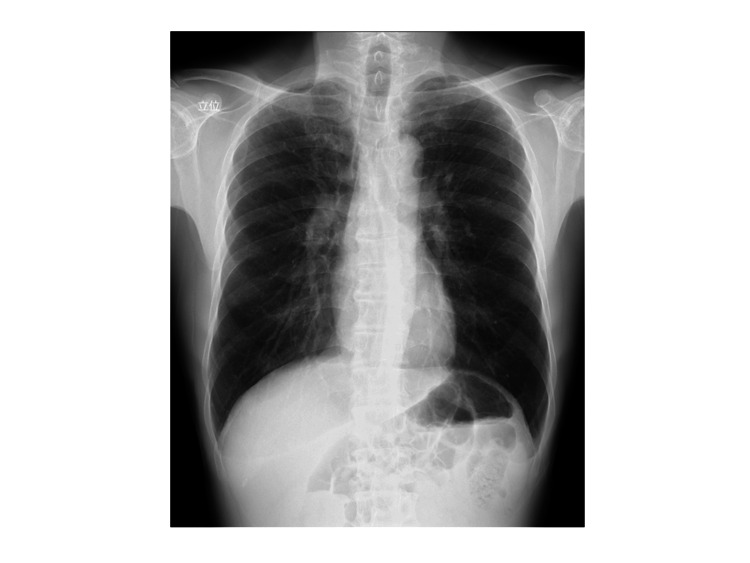
Chest radiography Chest radiography showed no abnormalities, including any that could be causing chest pain.

**Table 1:** Laboratory data at the initial presentation and day 1 (11 hours after the initial presentation) At the initial presentation, no abnormalities of cardiac biomarkers of myocardial necrosis were shown. Reexamination at 11 hours after the initial presentation revealed elevated cardiac biomarkers of myocardial necrosis, including creatine phosphokinase and troponin T.

Parameters	On the Initial Presentation	Day 1 (11 Hours after the Initial Presentation)	Reference Range
Hematocrit	39.5	38.1	40.7-50.1 %
Hemoglobin	13.7	13.1	13.7-16.8 g/dL
White cell count	7350	6160	3300-8600 /μL
Platelet count	173,000	180,000	156,000-348,000 /μL
C-reactive protein	0.05		0.00-0.14 mg/dL
Albumin	4.2		4.1-5.1 g/dL
Total cholesterol	206		142-248 mg/dL
Glucose	98		73-109 mg/dL
Glycated hemoglobin	6.1		4.9-6.0 %
Urea nitrogen	18.8	19.8	8-20 mg/dL
Creatinine	0.73	0.72	0.65-1.07 mg/dL
Uric acid	5.2	5.2	3.7-7.8 md/dL
Sodium	141	138	138-145 mmol/L
Potassium	4.1	4.1	3.6-4.8 mmol/L
Chloride	103	103	101-108 mmol/L
Creatine kinase	203	435	59-248 U/L
Creatine kinase MB	5		0-12 U/L
Lactate dehydrogenase	158	179	124-222 U/L
Aspartate aminotransferase	27	44	13-30 U/L
Alanine aminotransferase	17	18	10-42 U/L
Total bilirubin	0.5		0.4-15 mg/dL
Alkaline phosphatase	67		38-113 U/L
B-type natriuretic peptide	140.1		< 18.4 pg/mL
d-dimer	<0.5		0.0-1.0 μg/mL
Troponin T	Negative	Positive	Negative

Because the chest pain had continued at a rate of 7 on a scale of 0-10 (with 10 indicating the most severe pain), and the cause of the chest pain was not revealed, the responsible physician, who was a specialist in internal medicine but not in the subspecialty of cardiology, decided to admit the patient to the hospital under strict monitoring.

The next morning, cardiology was consulted to evaluate the patient. The chest pain had continued at a rate of 1 on a scale of 0-10. Re-examined ECG, obtained after 11 hours of having chest pain, showed no obvious ST-T changes compared with the initial ECG, except for T wave inversion in lead aVL (Figure 3).

**Figure 3:**
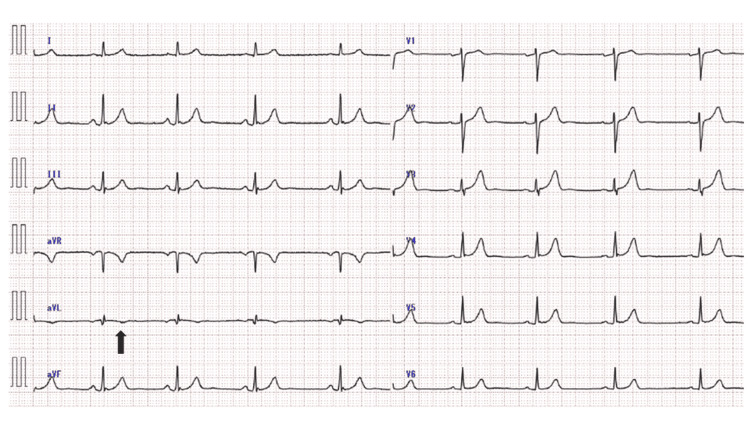
The next morning electrocardiogram (11 hours after AMI onset) Re-examined electrocardiogram obtained after 11 hours of having chest pain showed no obvious ST-T changes compared with the initial ECG (Figure 1), except for T wave inversion in lead aVL (black arrow). AMI denotes acute myocardial infarction.

Transthoracic echocardiography did not reveal any obvious abnormalities in left ventricular wall motion. Follow-up laboratory findings are shown in Table 1.

At that time, myocardial infarction was highly suspected because of troponin T becoming positive, and an emergent coronary angiogram was performed. An occlusion of the first diagonal branch was revealed, and a subsequent percutaneous coronary angioplasty using a 1.5 mm balloon was successfully performed, resulting in chest pain relief (Figure 4).

**Figure 4:**
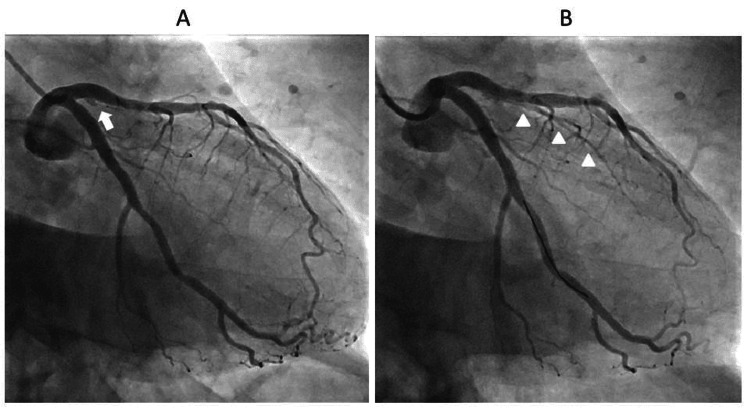
An emergent coronary angiogram An emergent coronary angiogram showed an occlusion of the first diagonal branch (Panel A, white arrow). After successful coronary angioplasty, TIMI3 flow was obtained in the first diagonal branch (Panel B, white arrow heads). TIMI denotes thrombolysis in myocardial infarction.

The maximum CPK was 631 U/L. The later clinical course was uneventful, and the patient was discharged from the hospital 10 days later. Follow-up ECGs did not show any significant changes afterward (Figures 5A-5C).

**Figure 5:**
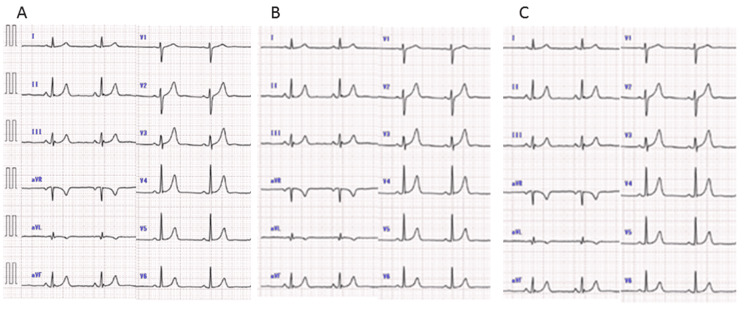
Electrocardiograms obtained four days, 10 days and 35 days after AMI onset A: Four days after AMI onset (three days after successful coronary angioplasty) B: Ten days after AMI onset (at discharge) C: Thirty-five days after AMI onset (at the outpatient clinic) Follow-up electrocardiograms showed no significant changes compared with the ECG obtained the next morning (Figure 3). AMI denotes acute myocardial infarction.

Later, we compared the initial ECG with a health checkup ECG obtained 14 months prior (Figure 6).

**Figure 6:**
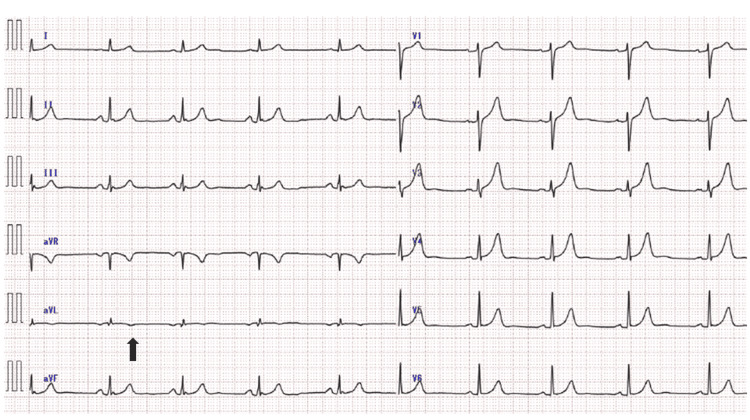
A health checkup electrocardiogram (14 months before AMI onset) A health checkup electrocardiogram obtained 14 months before AMI onset showed a negative T wave in lead aVL (black arrow), while the initial ECG showed a positive T wave (Figure 1). Hyperacute T waves seemed to appear in precordial leads on the initial ECG (Figure 1). However, comparing the initial ECG with the health checkup ECG and the ECG obtained 11 hours after onset (Figure 3), it is unlikely that these were hyperacute T waves because of little change in the T wave in precordial leads. AMI denotes acute myocardial infarction.

In read aVL, the ECG on the health check-up showed a negative T wave, while the initial ECG showed a positive T wave. Furthermore, a slight ST elevation in lead I could be interpreted on the initial ECG by comparison with the health checkup ECG.

## Discussion

This case involved a patient with a delayed diagnosis of AMI, despite early hospital arrival after onset. AMI is characterized by myocardial necrosis resulting from an acute occlusion of a coronary artery. Myocardial salvage by immediate reperfusion therapy, including percutaneous coronary angioplasty or fibrinolysis, is critical to limit myocardial necrosis; therefore, delayed diagnosis and treatment could lead to devastating consequences [3]. In our case, two reasons were considered to have contributed to the delayed diagnosis of AMI.

One reason is the subtle ECG changes on the initial ECG. A high lateral wall myocardial infarction caused by occlusion of the first diagonal branch, as in our case, is known that ECG changes could be less apparent [2]. This is because there are no leads that directly show this region, and the distance between the electrodes and this region is relatively far, resulting in small potential changes that are less noticeable in an ECG.

In our case, although the initial ECG obtained 50 minutes after AMI onset seemed to show no obvious ST-T changes, the ECG obtained 11 hours after onset showed a negative T wave in lead aVL only with no obvious change in other leads, and the health checkup ECG obtained 14 months prior also showed a negative T wave in lead aVL. Additionally, by comparison of the initial ECG with the health checkup ECG, ST elevation could be interpreted in lead I on the initial ECG. In this context, lead aVL on the initial ECG could be interpreted as ST elevation rather than an inverted T wave becoming positive, although it did not meet the diagnostic criteria for STEMI: 1mm ST-segment elevation in any two consecutive leads.

AMI has traditionally been classified into ST‑segment elevation myocardial infarction (STEMI) and non‑ST‑segment elevation myocardial infarction (NSTEMI). STEMI has historically been regarded as a surrogate for occlusion myocardial infarction (OMI), whereas NSTEMI has been considered a surrogate for non‑occlusion myocardial infarction (NOMI). However, approximately half of patients with OMI did not meet STEMI criteria, and 25% to 34% of NSTEMI cases had OMI, resulting in failure of timely reperfusion therapy. Consequently, a new concept of classification of myocardial infarction into OMI versus NOMI (based on pathophysiology) from STEMI versus NSTEMI (based on ST elevation) was proposed. The concept emphasizes whether immediate reperfusion therapy is truly necessary, rather than relying on the conventional STEMI/NSTEMI classification. In this context, ECG has become a key tool for detecting subtle changes suggestive of OMI, and several high‑risk OMI patterns have been identified [4].

In case of isolated first diagonal branch occlusion, 'hyperacute T wave' and 'South Africa flag sign' are known to be high-risk ECG patterns indicative of acute coronary occlusion [4]. In our case, hyperacute T waves appeared to be present in the precordial leads on the initial ECG. However, comparison with the health checkup ECG, as well as the ECGs obtained 11 hours and 35 days after AMI onset, showed minimal change in T‑wave morphology, making true hyperacute T waves unlikely (Figure 7).

**Figure 7:**
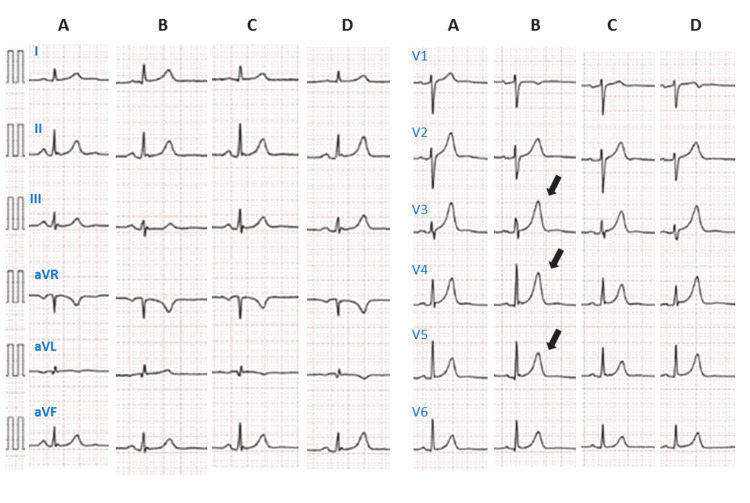
Electrocardiograms through the clinical course A: Heath checkup (14 months before AMI on set) B: At arrival (50 minutes after AMI onset) C: The next morning (11 hours after AMI onset) D: Thirty-five days after AMI onset (at the outpatient clinic) Hyperacute T waves appeared to be present in the precordial leads on the initial ECG (black arrow). However, comparison with the health checkup ECG, as well as the ECGs obtained 11 hours and 35 days after AMI onset, showed minimal change in T‑wave morphology, making true hyperacute T waves unlikely. AMI denotes acute myocardial infarction.

The South Africa flag sign is an ECG finding that shows ST elevation in leads I, aVL, and V2, and ST depression in lead III. The distribution of ST segment deviation resembles the pattern of the South African flag in a 3×4 lead display format of ECG. In our case, ST-T changes were only observed in leads I and aVL.

From our case, we realized again that it is important to compare an initial ECG with serial or prior ECGs to be aware of subtle ECG changes in order not to miss or delay the diagnosis of AMI, when it has not been ruled out.

Another reason for the delayed diagnosis is that the cardiac biomarker troponin T was negative at presentation. The 2025 ACC/AHA/ACEP/NAEMSP/SCAI guideline for the management of patients with acute coronary syndrome recommends repeat measurements of conventional troponin T at 3 to 6 hours after initial sampling when acute coronary syndrome has not been ruled out [5]. Although our patient arrived at the hospital just 50 minutes after the onset, AMI was not immediately diagnosed at the time because the initial troponin T was within the normal range. Positive reexamination of troponin T 11 hours after onset led to a high suspicion of AMI; subsequently, an emergent coronary angiogram revealed an occlusion of the first diagonal branch, and percutaneous coronary angioplasty was performed. It had consumed time for diagnosing AMI. If troponin T had been followed up three to six hours after arrival, the result could have become positive, and AMI could have been diagnosed earlier. Despite our patient’s clinical course being uneventful, we realized the importance of serial cardiac biomarker measurements when AMI has not been ruled out.

## Conclusions

We report a case of a high lateral AMI caused by occlusion of a first diagonal branch with subtle ECG changes and negative cardiac biomarkers at presentation, resulting in a delayed diagnosis. We realized that it is important to compare an initial ECG with serial or prior ECGs to be aware of subtle ECG changes and to repeat cardiac biomarker measurements a few hours later in order not to miss or delay the diagnosis of AMI, when it has not been ruled out.
